# Blocking Intermediate-Conductance Calcium-Activated Potassium Channels in the Macrophages Around Ganglionated Plexi Suppresses Atrial Fibrillation Vulnerability in Canines With Rapid Atrial Pacing

**DOI:** 10.3389/fphys.2022.837412

**Published:** 2022-04-01

**Authors:** Yazhe Ma, Yuntao Fu, Youcheng Wang, Mei Yang, Yajun Yao, Shanqing He, Dishiwen Liu, Zhen Cao, Xi Wang, Yanhong Tang, Qingyan Zhao, Congxin Huang

**Affiliations:** ^1^ Department of Cardiology, Renmin Hospital of Wuhan University, Wuhan, China; ^2^ Cardiovascular Research Institute of Wuhan University, Wuhan, China; ^3^ Hubei Key Laboratory of Cardiology, Wuhan, China

**Keywords:** atrial fibrillation, intermediate-conductance calcium-activated potassium channels, ganglionated plexi, macrophage polarization, inflammatory cytokine

## Abstract

Previous studies have indicated that ganglionated plexi (GP) function influences atrial fibrillation (AF) vulnerability, and intermediate-conductance calcium-activated potassium channels (SK4) have a close relationship with cardiomyocyte automaticity and the induction of AF. However, the effects of the SK4 inhibitor on GP function and AF vulnerability are unknown. Eighteen beagles were randomly divided into a control group (n = 6), rapid atrial pacing (RAP) group (n = 6), and triarylmethane-34 (TRAM-34, an SK4 inhibitor) group (n = 6). TRAM-34 (0.3 ml, 15 mmol/L) and saline were locally injected into GPs in the TRAM-34 group dogs and dogs from the other groups, respectively. After that, dogs in the RAP and TRAM-34 groups were subjected to RAP, and the neural activity of anterior right GP (ARGP) and atrial electrophysiology were measured. The levels of inflammatory cytokines and function of macrophages in the ARGP were measured in the three groups. At 10 min after TRAM-34 injection, ARGP activity and atrial electrophysiology did not significantly change. The atrial pacing shortened effective refractory period (ERP) values at all sites and increased the AF vulnerability and ARGP neural activity, while TRAM-34 reversed these changes. The levels of CD68 ^+^ cells, induced nitric oxide synthase (iNOS), interleukin (IL)-1β, IL-6, and tumor necrosis factor (TNF)-α in the ARGP tissues were higher in the RAP group and TRAM-34 group than they were in the control group. Furthermore, the levels of the CD68 ^+^ cells, iNOS, and inflammatory cytokines in the ARGP tissues were higher in the pacing group than those in the TRAM-34 group. Based on these results, administration of TRAM-34 into the atrial GP can suppress GP activity and AF vulnerability during atrial pacing. The effects of TRAM-34 might be related to macrophage polarization and the inflammatory response of GP.

## Introduction

Atrial fibrillation (AF) is the most common clinical arrhythmia in the elderly and causes severe complications such as stroke, amentia, cardiac insufficiency, and high rates of hospitalization (Rahman et al., 2014; Staerk et al., 2017). Previous studies have demonstrated that ganglionated plexi (GP), which mainly constitute the intrinsic cardiac nerve system (ICANS), play an important role in the initiation and maintenance of AF, and interfering GP activity may suppress AF vulnerability (Mao et al., 2012; Lo et al., 2013; Malcolme-Lawes et al., 2013; Lu et al., 2015). Recently, Yu found that the inflammation status of fat pads can influence GP activity and AF vulnerability (Yu et al., 2018). The increased inflammatory cytokines in the GP may aggravate the acute atrial electrical remodeling in a rapid atrial pacing (RAP)-induced AF model.

Recent studies have shown that intermediate-conductance KCa channel (SK4, KCa3.1) play a key role in arrhythmia (Haron-Khun et al., 2017; Bueno-Levy et al., 2020). Our previous studies indicated that SK4 is closely related to AF vulnerability, and the expression of SK4 in the atrium is related to stellate ganglion activity during rapid atrial pacing (Yang et al., 2020; Yang et al., 2021). It has been demonstrated that blocking SK4 can inhibit neuroinflammation in the nerve system, and SK4 can regulate pro-inflammatory phenotype change in macrophages (Staal et al., 2017; Xu et al., 2017; Lu et al., 2019). However, the effects of blocking SK4 in the GP on GP function and AF vulnerability have not been reported. Therefore, the purpose of this study was to investigate the effects of SK4 inhibitor (triarylmethane-34, TRAM-34) on GP function and AF vulnerability during RAP.

## Materials and Methods

This study was approved by the animal studies subcommittee of our institutional review board (WDRM 20191211) and was in compliance with the guidelines of the National Institutes of Health for the care and use of animals in an experiment.

### Animal Model Preparation

Eighteen adult beagles, weighing an average of 8.6 ± 1.5 kg, were used in this study. Each beagle was administered an intramuscular injection of 25 mg/kg ketamine sulfate before being premedicated with pentobarbital sodium (30 mg/kg intravenously), intubated, and ventilated with room air supplemented with oxygen by a respirator (MAO01746, Harvard Apparatus Holliston, United States). An extra pentobarbital sodium injection (6 mg/kg, every 2 h) was administered during the experiment to keep the animal anesthetized. Normal saline (50–100 ml/h) was infused to replace spontaneous fluid loss. Continuous electrocardiogram was monitored by Lead 7000 20 Lab System (Jinjiang Inc., Chengdu, China).

### Experimental Protocol

**FIGURE 1:**
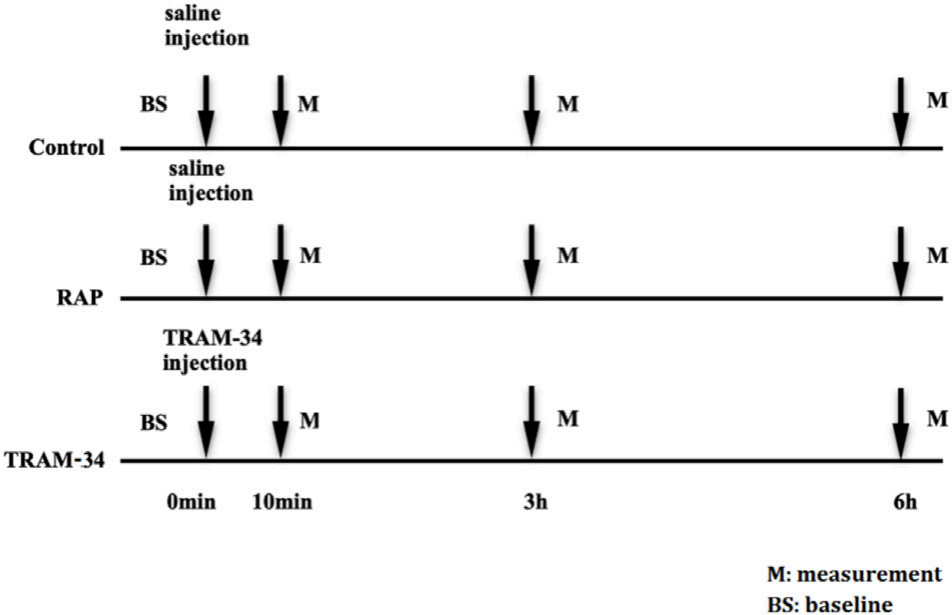
Experimental flow chart. Abbreviations: BS, baseline; RAP, rapid atrium pacing; M, measurement.

The dogs were randomly divided into three groups. The control group consisted of six dogs that underwent saline injection into four major GP (the anterior right GP (ARGP), the inferior right GP, the superior left GP, and the inferior left GP) without RAP. The RAP group consisted of six dogs that underwent saline injection into four major GP plus 800 beats/min RAP for 6 h. The TRAM-34 (HY-13519, Med Chem Express, United States) group consisted of six dogs that underwent TRAM-34 injection into GP combined with RAP for 6 h. The ARGP neural activity and electrophysiological properties were determined at different times. The voltage we used in the RAP is 4 V. The protocol is shown in Figure 1.

### GP Injection

All dogs received bilateral thoracotomy at the fourth intercostal space to optimize GP injection under inspection. Before RAP, the GP were identified depending on the anatomical landmarks and heart rate slowing phenomenon under high-frequency stimulation. High-frequency electrical stimulation (20 Hz, 0.1 ms duration, square waves, 0.6–6.0 V) was performed using a bipolar electrode probe through an electrophysiology stimulator (SEN-7103, Japan). Vagal reflex was defined as a ≥50% increase in mean R–R interval or when complete atrio-ventricular (AV) block occurred. The TRAM-34 (0.3 ml, 15 mmol/L) was locally injected into the four GP in the TRAM-34 group, and saline in the same volume was locally injected into the four GP in the control and RAP groups.

### Measurement of Electrophysiological Properties

Ten minutes after GP injection of TRAM-34 or saline, effective refractory period (ERP) in the right atrial appendage (RAA), left atrial appendages (LAA), right superior vein (RSPV), right inferior vein (RIPV), left superior vein (LSPV), and left inferior vein (LIPV) were determined. An S1S1 stimulus method (120, 100, and 75 ms cycle length, each cycle stimulated three times, lasting for 5 s) was adopted to assess the inducibility and duration of AF. The AF was defined as an irregular atrial rate >500 bpm lasting for more than 5 s. Electrophysiological properties were measured at baseline, 3 and 6 h after RAP.

### Measurement of GP Activity

**FIGURE 2:**
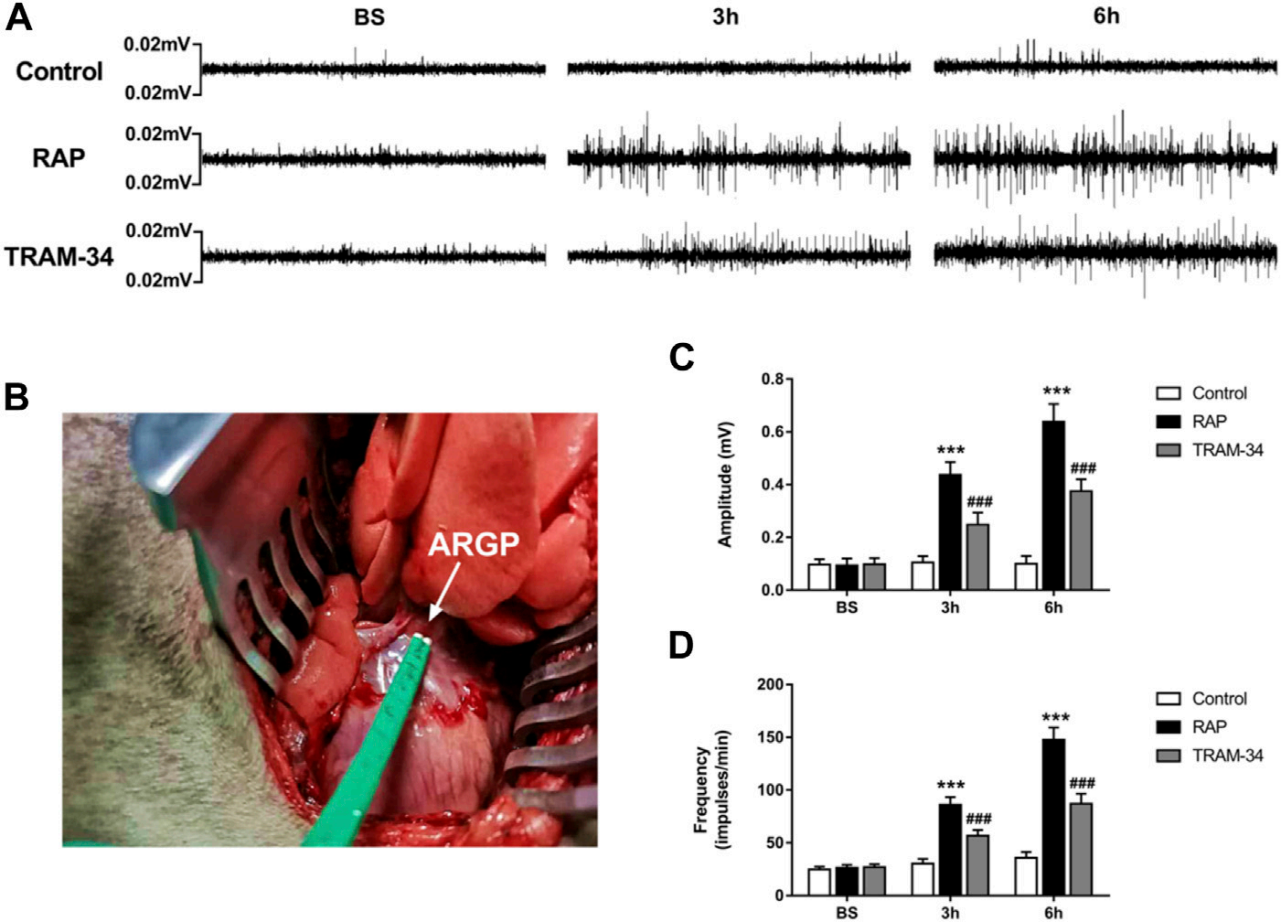
ARGP activity at baseline and after rapid atrial pacing in the three groups. **(A)** ARGP activity at baseline, after 3 h, and after 6 h in the three groups; **(B)**: Microelectrodes were inserted into the ARGP; **(C,D)** The frequency and the amplitude of ARGP in the three groups. The frequency and amplitude of ARGP activity was increased after rapid atrial pacing. TRAM-34 injection inhibited the increased ARGP activity. ***p < 0.01vs the control group and TRAM-34 group; ###*p* < 0.01vs the control group.

Microelectrodes (Xi’an Friendship Medical Electronics Co. Ltd., China) were inserted into the epicardial fat pad surrounded by the superior vena cava and right atrium to measure ARGP neural activity (Figure 2B). The neural signals were acquired and amplified by Power Lab system (AD Instruments, Dunedin, New Zealand) at baseline and after GP injection (10 min, 3 h, and 6 h after injection) in the pacing and TRAM-34 groups. Neural signals that were three times higher than the noise signal were recognized as neural activity. The frequency and amplitude of the neural discharge during this period were calculated automatically by Lab Chart 7 software (AD Instruments, Dunedin, New Zealand).

### Immunofluorescence

At the end of the measurement, the animals were euthanized by overdose anesthesia. The ARGP tissues were quickly removed from the heart, fixed in 4% paraformaldehyde, and embedded in paraffin. Tissues were cut into 5-μm sections. The samples were incubated with primary antibodies against CD68 (1:100, Abcam, United Kingdom) and subsequently with secondary antibodies (FITC-conjugated Affinipure Goat Anti-rabbit IgG). Cell nuclei were stained with DAPI. Image Pro Plus 6.0 (Media Cybernetics, Inc., Rockville, Maryland) was used to quantitative analysis; CD 68 + cell area was equal to the CD68 positive area divided by the whole area of selected field. Three visual fields at 400X were randomly tested from each sample.

### Real-Time Polymerase Chain Reaction (RT-PCR)

The expression of induced nitric oxide synthase (iNOS) and arginase-1(Arg-1) in ARGP was measured by RT-PCR. Total RNA was isolated from ARGP samples with Tripure Extraction Reagent (ELK Biotechnology, China) according to the manufacturer’s protocol. Next, cDNA was synthesized using an EntiLink™ first Strand cDNA Synthesis Kit (ELK Biotechnology, China). The 2^–ΔΔCt^ method was used to calculate the mRNA values of iNOS and Arg-1. The primer sequences and amplicon sizes of the selected genes are shown in Table 1.

**TABLE 1:** Primer sequences and amplicon sizes of genes validated by TaqMan™ RT-PCR.

Gene Name	Accession No	Primer sequence (5′-3′)		Amplicon Size, bp^a^
GAPDH	NM_001003142.2	sense	GAA​GGT​CGG​AGT​GAA​CGG​ATT	249
		antisense	CAT​TTG​ATG​TTG​GCG​GGA​TC	
inos	NM_001313848.1	sense	ACC​AAT​ACA​GGC​TCG​TGC​AG	265
		antisense	GGG​CTG​TCT​ACT​ACT​CGC​TCC	
Arg-1	XM_532053.6	sense	GGC​AGA​AGT​CAA​GAA​GAA​CGG	266
		antisense	CTT​TGG​CAG​ATA​GGC​AAG​GAG	

a bp, base pairs.

### Western Blot Analysis

Total protein was extracted from the ARGP. Western blotting was performed using the procedure recommended in previous study (Yang et al., 2020). Primary antibodies utilized for incubation were anti interleukin (IL)-1β (1:10,000, Bioss, United States), anti IL-6 (1:1,000, R&D, United States), and anti tumor necrosis factor (TNF)-α (1:1,000, LSBio, United States). After incubation with the antibodies overnight at 4°C, the membranes were washed three times. Then they were incubated with the secondary antibody for 2 h at room temperature. The proteins were normalized to β-Actin (1:5,000, Abcam, United States). The blot images were scanned and analyzed using image analyzer software (AlphaEase FC, United States).

### Statistical Analysis

All continuous data were presented as the mean ± standard deviation. Paired T-tests were used to compare ERP and neural activity changes within groups. One-way ANOVA followed by Bonferroni post-hoc tests were used to compare the values of neural activity; AF inducibility and duration; and the values of IL-1β, IL-6, and TNF-α; and iNOS+, arg-1+, and CD68 ^+^ cell area among three groups. Values were considered significant at *p* < 0.05.

## Results

### Neural Activity of ARGP

The neural activity of the ARGP was recorded at baseline, at 10 min after GP injection, and at 3 and 6 h of pacing. The results are shown in Figures 2A,C. In the RAP group, the ARGP nerve activity was significantly increased at 3 and 6 h when compared to that at the baseline time (*p* < 0.05). For example, compared with those at baseline, the frequency and amplitude of the ARGP were significantly increased after 3 h pacing (frequency: 26.3 ± 2.9 versus 86.0 ± 7.4 impulses/min, *p* < 0.001; amplitude: 0.09 ± 0.05 versus 0.44 ± 0.05 mV, *p* < 0.001). In the TRAM-34 group, there was no significant difference in the frequency (26.8 ± 3.2 impulses/min vs 28.6 ± 5.1 impulses/min; *p* > 0.05) or amplitude (0.10 ± 0.02 vs. 0.12 ± 0.05 mV; *p* > 0.05) of ARGP activity before vs 10 min after TRAM-34 injection. After 3 and 6 h, the frequency and amplitude of the ARGP were significantly increased when compared with those at baseline. The frequency and amplitude of the ARGP were much higher in the pacing group compared with the control group after 3 and 6 h (*p* < 0.001 for all). However, compared with those in the pacing group, the frequency and amplitude of the ARGP were lower in the TRAM-34 group after RAP. For example, the frequency and amplitude of the ARGP were 56.7 ± 5.6 impulses/min and 0.24 ± 0.05 mV in the TRAM-34 group, and they were 86.0 ± 7.4 impulses/min and 0.44 ± 0.05 mV in the pacing group at 3 h (*p* < 0.001 for all).

### ERP and AF Vulnerability

**FIGURE 3:**
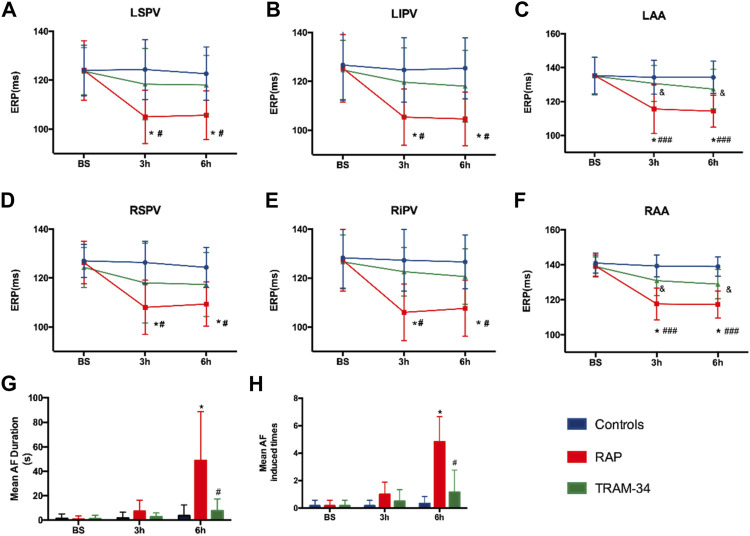
Results of electrophysiological measurements. **(A–F)** ERP changes at RAA, LAA, RSPV, RIPV, LSPV, and LIPV during 6-h stimulation. *p < 0.05 vs baseline **(G)** Mean AF duration, and **(H)** Mean AF inducibility changes in the Control, RAP, and TRAM-34 groups. *p < 0.05 vs baseline; ^#^ *p* < 0.05 vs the RAP group. Abbreviations: RAA, right atrial appendage, LAA, left atrial appendage RSPV, right superior pulmonary vein, RIPV, right inferior pulmonary vein, LSPV, left superior pulmonary vein, LIPV, left inferior pulmonary vein, ERP, effective refractory period, AF, atrial fibrillation.

In the RAP group, the ERP at all recording sites was significantly shortened during rapid atrial pacing. For example, the AERP at the RA site was shortened from 139 ± 6.1 ms at the baseline state to 117 ± 5.8 ms after 3 h and reached 117 ± 7.3 ms at 6 h (*p* < 0.05). In the TRAM-34 group, the ERP also decreased during RAP. For example, the AERP at the RA site was shortened from 139 ± 5.5 ms at the baseline state to 131 ± 8.5 ms at 3 h and reached 129 ± 8.6 ms at 6 h (*p* > 0.05), but this was not statistically significant (Figure 3).

**FIGURE 4:**
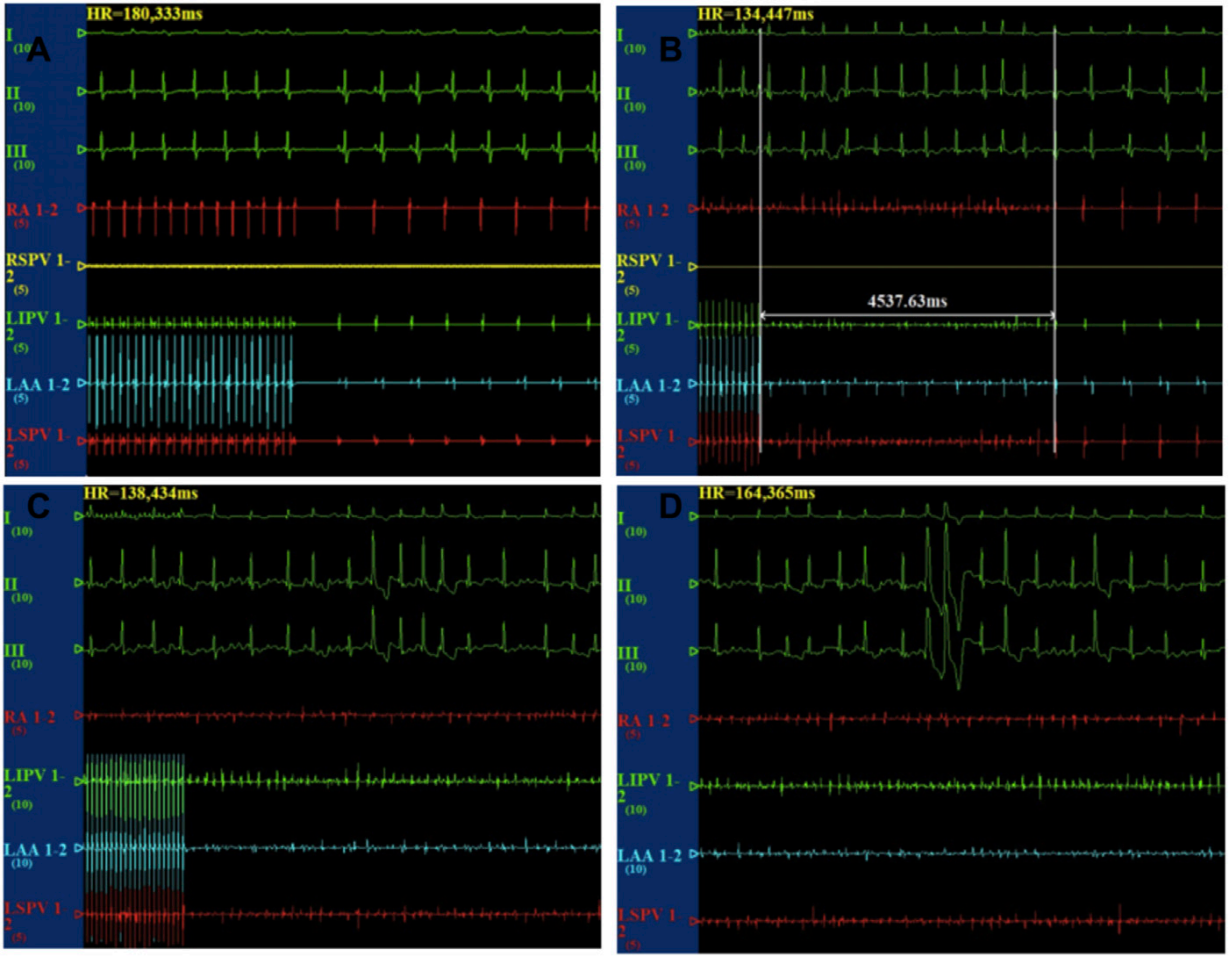
Representative picture of AF induction in the three groups. In the control group, the burst pacing didn’t induce AF **(A)**. In the TRAM-34 group, after 6 h pacing, the burst pacing didn’t induce AF **(B)**. In the RAP group, after 6 h pacing, the burst pacing induced AF last for 21.3 s **(C,D)**.

After the cessation of 6 h pacing, the mean AF times (4.8 ± 1.7 vs 0.17 ± 0.4, *p* < 0.001) and the AF duration (48.9 ± 39.9 vs 1.1 ± 2.7 s, *p* < 0.05) were higher in the pacing group when compared with the baseline. However, there was no change in the mean AF times (0.17 ± 0.4 vs 1.2 ± 2.9 times, *p* > 0.05) and the AF duration (1.0 ± 2.4 vs 7.6 ± 9.7 s, *p* > 0.05) between the baseline and after 6 h pacing in the TRAM-34 group. The mean AF times (0.33 ± 0.81 vs. 4.8 ± 1.7 times, *p* < 0.05) and AF durations (3.6 ± 8.6 vs 48.9 ± 39.9s) were increased in the RAP group compared with control group. The mean AF times (1.2 ± 2.9 vs 4.8 ± 1.7s, *p* < 0.05) and AF durations (7.6 ± 9.7 vs 48.9 ± 39.9 s, *p* < 0.05) were lower in the TRAM-34 group compared those with the RAP group. The representative ECG pictures were shown in Figure 4.

### Macrophage Phenotype

Immunostaining was performed to assess macrophages by quantifying CD68^+^, and PCR was performed to assess the macrophage subtypes: classically activated macrophage (M1), or alternatively activated macrophage (M2) using a specific marker (M1: iNOS+, M2: arg-1+).

**FIGURE 5:**
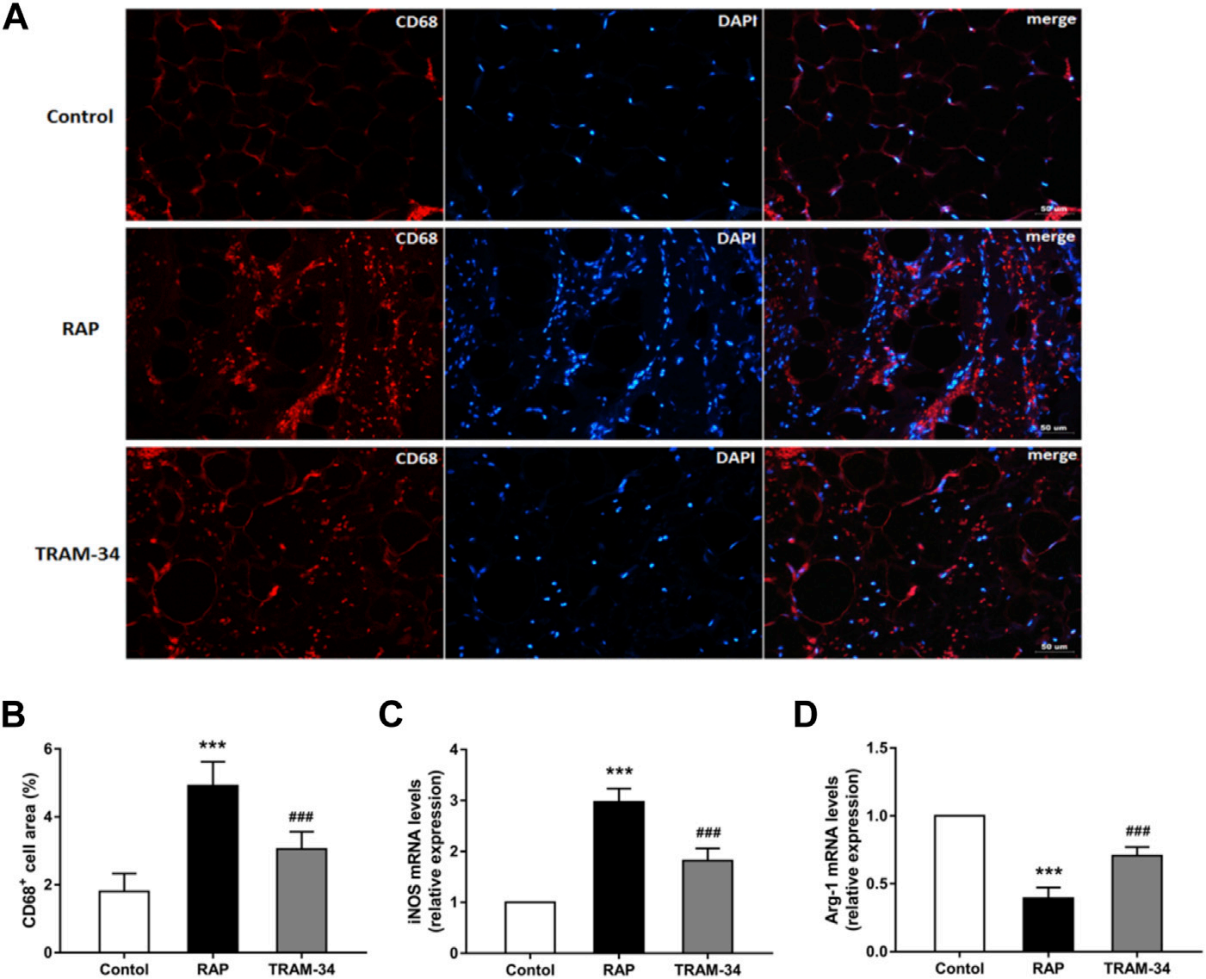
Levels of CD68^+^, iNOS, and arg1 in the ARGP in the three groups. Representative images and quantitative analysis of immunofluorescence staining for CD68^+^ (red) in the ARGP **(A,B)** (n = 6, 40X). Quantitative analysis of RT-PCR of iNOS and arg1 in ARGP **(C,D)**. RAP increased the secretion of inflammation cytokines and promoted macrophage polarization from M2 to M1 compared to that in the control group. However, TRAM-34 can attenuate this effect. ***p < 0.01vs the control group and TRAM-34 group; ###*p* < 0.01vs the control group.

As shown in Figure 5, the CD68 ^+^ cell areas in the ARGP tissues were significantly higher in the pacing group than in the control group (CD68+: 4.9 ± 0.7% vs 1.8 ± 0.5%, *p* < 0.001) and TRAM-34 group (CD68+: 4.9 ± 0.7% vs 3.1 ± 0.5%, *p* < 0.001). Furthermore, the levels of the CD68 ^+^ cell in the ARGP tissues were significantly higher in the TRAM-34 group than in the control group (CD68+: 3.1 ± 0.5% vs. 1.8 ± 0.5%, *p* < 0.001). The levels of iNOS were significantly higher in the pacing group than in the control group (*p* < 0.001) and TRAM-34 group (iNOS: 2.97 ± 0.27 vs 1.82 ± 0.25, *p* < 0.001). The levels of arg-1 were significantly lower in the pacing group than in the control group (*p* < 0.001) and TRAM-34 group (0.39 ± 0.07 vs 0.71 ± 0.07, *p* < 0.001).

### Inflammatory Cytokines

**FIGURE 6:**
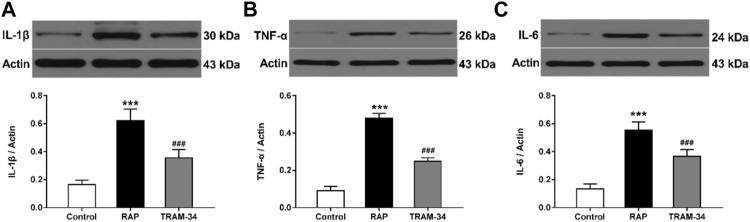
Levels of inflammatory cytokines in the three groups. Western blotting and quantitative analysis of IL-1β, TNF-α, and IL-6 in ARGP **(A–C)**. ***p < 0.01 vs the control group and TRAM-34 group; ###*p* < 0.01vs the control group.

As shown in Figure 6, the levels of IL-1β, IL-6, and TNF-α in the ARGP tissues were significantly higher in the RAP group when compared with the control group (IL-1β: 0.62 ± 0.07 vs 0.16 ± 0.02, IL-6: 0.55 ± 0.05 vs 0.16 ± 0.02, TNF-α: 0.48 ± 0.02 vs 0.09 ± 0.02, all *p* < 0.001), and the TRAM-34 group (IL-1β: 0.62 ± 0.07 vs 0.36 ± 0.05, IL-6: 0.55 ± 0.05 vs 0.37 ± 0.05, TNF-α: 0.48 ± 0.02 vs 0.25 ± 0.02; *p* < 0.001). Furthermore, the levels of IL-1β, IL-6, and TNF-α in the ARGP tissues were significantly higher in the TRAM-34 group when compared with the control group (IL-1β: 0.36 ± 0.05 vs 0.16 ± 0.02, *p* < 0.01; IL-6: 0.14 ± 0.02 vs 0.37 ± 0.05, *p* < 0.01; TNF-α: 0.09 ± 0.02 vs 0.25 ± 0.02, *p* < 0.01).

## Discussion

### Analysis of Findings

This study explored the influence of TRAM-34 on GP and AF vulnerability after experimental pacing in canines. We provide evidence for the following: 1) TRAM-34, an SK4 channel antagonist, has no effect on the GP neural activity, atrial electrophysiology properties, and AF vulnerability via direct injection in GP. 2) TRAM-34 attenuates GP neural activity and AF vulnerability in the rapid pacing canine model. 3) TRAM-34 inhibits macrophages polarization to M1 and decreases the levels of inflammation factors in GP.

The GP neural clusters around pulmonary veins contain parasympathetic neurons and sympathetic afferents and efferents. Evidence from experimental and clinical studies has shed light on the role of GP in the initiation and maintenance of AF (Hanna and Shivkumar, 2018). It is possible that GP hyperactivity induces the secretion of neurotransmitters such as acetylcholine and catecholamines, which facilitate decreasing ERP and increasing AF vulnerability. In addition, GP ablation could reverse electrical remodeling and neural remodeling induced by rapid pacing (Zhou et al., 2007; Avazzadah et al., 2020). Studies have shown that KCa channels are present in nerve cells and responsible for after-hyperpolarization that suppresses nerve discharges, playing a vital role in regulating sympathetic nerve activities (Samengo et al., 2014; Sforna et al., 2018). Activation of KCa can inhibit nerve activity, while blocking KCa could increase nerve activity (Moraes et al., 2014). In our study, we injected TRAM-34 directly into four major atrial GP and found that TRAM-34 injection alone had no effects on the neural firings in ARGP and atrial electrophysiology did not cause significant change. These results showed that the dose of TRAM-34 is not enough to cause GP activity and atrial electrophysiological changes. In the present study, we further found that GP activity significantly increased and AF was induced easily after RAP, while TRAM-34 suppressed the GP activity and AF vulnerability during RAP. Ablation of GP of the intrinsic cardiac nerves with catheter ablation methods may be effective in inhibiting AF (Lu et al., 2008). However, GP ablation may not achieve long-term suppression of AF induction in the canine model (Oh et al., 2006). Clinical evidence shows that GP ablation actually have no benefit on AF suppression in long term (Kim et al., 2021). Increased concentrations of inflammatory factors in the atrium after GP ablation provide a new causative factor in terms of AF vulnerability (Zhao et al., 2011). Consistent with the previous study, our results indicated that the effects of TRAM-34 injection in GP on AF vulnerability during RAP are closely related to the GP activity.

Inflammation has been demonstrated to regulate nerve activities, which are important in arrhythmia initiation and maintenance (Conte et al., 2022). Previous study showed that injection of IL-1β into the left stellate ganglion led to increased sympathetic nerve activity (Wang et al., 2017). Inflammatory status in the fat pad has been demonstrated to regulate GP nerve activity, and nerve activity is important in the initiation and maintenance of arrhythmia. Previous studies have shown that injection of inflammatory mediators (trimethylamine N-oxide, adipokine) into GP could increase GP nerve activity and AF vulnerability (Yu et al., 2018). In our study, we found that the levels of inflammatory cytokines such as IL-1β, IL-6, and TNF-α in ARGP were increased after 6 h pacing in the RAP group, while TRAM-34 injection in GP suppressed the increased pro-inflammatory cytokines after atrial pacing. Furthermore, we found that TRAM-34 suppressed macrophage polarization from M2 to M1, which likely accounted for the reduction in inflammatory cytokines in the ARGP. Previous studies have demonstrated that M1 macrophages produce pro-inflammatory cytokines (IL-1β, IL-6, and TNF-α) while M2 macrophages mainly exert an anti-inflammatory effect (Biswas et al., 2008). Inflammatory cytokines secreted by M1 macrophages were increased in AF patients, and promoting macrophages polarization from M1 to M2 could suppress AF (Guo et al., 2012; Liu et al., 2019). It has been confirmed that the SK4 inhibitor significantly reduced the expression of pro-inflammatory genes and promoted macrophage polarization from M1 to M2 (Xu et al., 2017). Our findings suggest that GP neural activity was increased under the pro-inflammatory environment during RAP. The SK4 inhibitor suppressed macrophage polarization from M2 to M1 and decreased inflammatory cytokines during RAP. These results may explain why the TRAM-34 had different effects on ARGP nerve activity with or without RAP. Finally, TRAM-34 inhibited AF vulnerability through suppressing inflammatory reaction directly in GP.

### Clinical Implications

Atrial fibrillation is the most common cardiac arrhythmia, affecting over 33 million people worldwide, and the incidence of AF is increasing (Rahman et al., 2014). However, there has been no progress in the development of new antiarrhythmic drugs in recent years. The SK4 channel is expressed in a limited way in the atrium, nerves, and macrophages. In the present study, we demonstrated that TRAM-34, a SK4 inhibitor, inhibited inflammatory cytokines in GP and suppressed AF vulnerability in the RAP-induced canine model. Our findings provide an experimental basis for clinical AF control by targeting SK4 in GP and may be a new promising pathway for AF control in the future.

### Study Limitations

This study has several limitations. First, TRAM-34 was administered as a local injection to observe GP activity and AF vulnerability. The dose was based on a previous study, but we did not investigate the effect of different concentrations of TRAM-34 on the GP. Second, in our previous study, we found that rapid atrial pacing could increase the expression of SK4 in atrium. In this study, we did not test the expression of SK4 in the three groups. A previous study showed that TRAM-34 suppresses T-cell mitogenesis (Wulff et al., 2000). However, we did not investigate the effect of TRAM-34 on T-cells in GPs. Whether the reduction in inflammatory mediators after TRAM-34 injection has a correlation with T-cells is unknown. Furthermore, we did not further distinguish circulating monocytes and cardiac resident macrophage types.

## Conclusion

This study demonstrates that local administration of TRAM-34 into the atrial GP can suppress GP activity and AF vulnerability during RAP. The effects of TRAM-34 may be related to the macrophage polarization and inflammatory response of GP.

## Data Availability

The original contributions presented in the study are included in the article/Supplementary Material, further inquiries can be directed to the corresponding authors.
