# Supplementary figures and images for: Blocking Intermediate-Conductance Calcium-Activated Potassium Channels in the Macrophages Around Ganglionated Plexi Suppresses Atrial Fibrillation Vulnerability in Canines With Rapid Atrial Pacing
Source: Front Physiol. 2022 Apr 1;13:837412. doi: 10.3389/fphys.2022.837412 (PMC9010666; doi:10.3389/fphys.2022.837412)

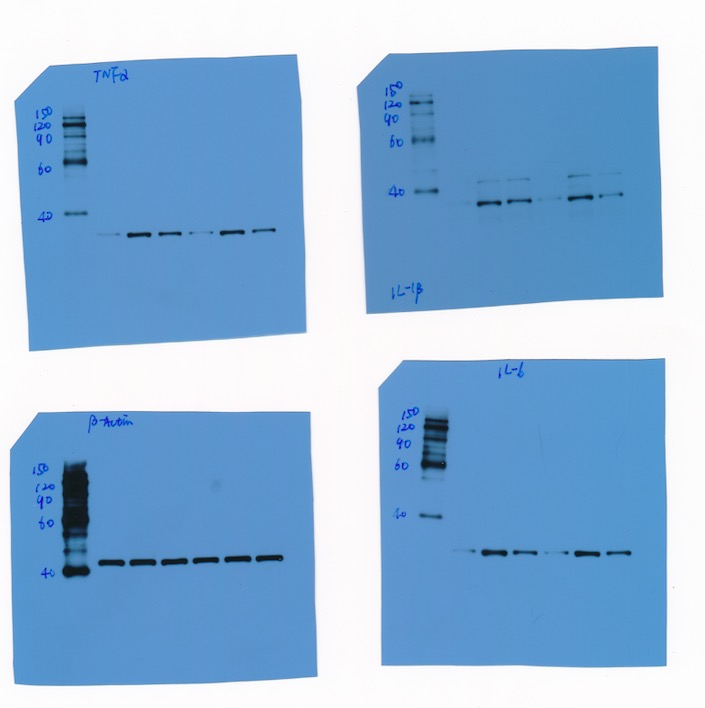

Supplement: Supplementary file 1 [file Image1.JPEG]
